# Clinical and genomic evaluation of a Chinese patient with a novel deletion associated with Phelan–McDermid syndrome

**DOI:** 10.18632/oncotarget.12552

**Published:** 2016-10-10

**Authors:** Dongzhu Lei, Shaoyuan Li, Santasree Banerjee, Haoqing Zhang, Caiyun Li, Shuai Hou, Danjing Chen, Haiying Yan, Hanmei Li, Huan Huan peng, Saijun Liu, Xinxin Zhang, Zhiyu Peng, Jian Wang, Huanming Yang, Hui Huang, Jing Wu

**Affiliations:** ^1^ Chenzhou No.1 People's Hospital, Chenzhou, China; ^2^ BGI-Shenzhen, Shenzhen, China; ^3^ Changsha Maternal and Child Health Hospital, Hunan, China; ^4^ BGI Education Center, University of Chinese Academy of Sciences, Shenzhen, China; ^5^ James D. Watson Institute of Genome Sciences, Hangzhou, China

**Keywords:** Phelan–McDermid syndrome, 22q13 deletion syndrome, novel deletion, SHANK3, translocation

## Abstract

Phelan–McDermid syndrome is a neurodevelopmental disorder caused by the terminal deletion of chromosome 22 (22q13) followed by the loss of function of the *SHANK3* gene. Various terminal deletions of chromosome 22q13 are associated with Phelan–McDermid with a spectrum of phenotypic severity. Here, we have done a clinical molecular study of a Chinese proband with Phelan–McDermid syndrome. Both the proband and her younger brother are associated with this syndrome while their parents are phenotypically normal. We used a karyotype in order to detect the genotype of the proband and her younger brother. We have also used whole genome low-coverage paired-end next generation sequencing to determine whether the parent is the carrier of translocation with terminal 22q13 deletions. We found that both proband and her younger brother are comprises of a novel deletion of 22q13.31q13.33, harboring genes were associated with several clinical phenotype such as severity of speech delay, neonatal hypotonia, delayed in age of walking, male genital anomalies, dysplastic toenails, large and fleshy hands, macrocephaly, short stature, facial asymmetry, and atypical reflexes. Probands and her younger brother inherited this translocation from their mother whereas their father is genotypically normal. In conclusion, our present study expands the deletion spectrum and report a novel deletion associated with Phelan–McDermid syndrome.

## INTRODUCTION

Phelan–McDermid syndrome (PMS) [MIM# 606232] is a rare neurodevelopmental disorder caused by terminal deletions or rearrangements of the long (q) arm of the chromosome 22. Phelan–McDermid syndrome is manifested with global developmental delay, delayed speech, neonatal hypotonia, autistic-behaviors, and mild dysmorphic features [1]. Neurological symptoms of PMS is associated with deletion of *SHANK3* (SH3 and multiple ankyrin repeat domains 3) gene, located in the 22q13.33 [2, 3]. In most of the PMS patients, deletion of IB2 (islet-brain 2) gene has been reported. IB2 gene is located 70 kb proximal to *SHANK3*. Based on previous report, IB2 may significantly associate with synaptic stability and neuronal transmission [4]. Amongst all the PMS patients, the chromosomal deletions are ranging from 100 kb to over 9 Mb [5]. This syndrome is mainly caused by deletion of *SHANK3* (*PSAP2*) gene. Deletions ranged from 277 kb to 4.36 Mb in *SHANK3* gene was observed in 3 of 400 autism spectrum disorder patients [6].

*SHANK3* gene is expressed in a highly tissue-specific manner. Specifically, in brain tissues, *SHANK3* gene is highly expressed and the expression is regulated by tissue-specific methylation [7, 8]. It is a structural protein, significantly functions at excitatory synapses in the brain by the assembly, maintenance and plasticity of the postsynaptic density (PSD) [9]. SHANK3 gene has been found to be associated with PMS along with other PSD components, including cell adhesion molecules such as neurexins and neuroligins [10-15]. Recently Wilson et al. reported that PMS phenotypes are also associated with interstitial deletions outside of the SHANK3 region [16].

In the present study, karyotype and whole genome low-coverage next generation sequencing has undertaken in order to assess the copy number variants and the structure variants for a proband of Chinese descent has been suffering from developmental delay, severely delayed speech, autistic like behaviors, and mild dysmorphic features. Proband and her younger brother were identified with 22q13 deletion syndrome by unbalanced translocation, inherited from balanced translocation t(9;22)(q34.3;q13.31) from their mother. Karyotype failed to detect the small CNV on 9q34.3, so whole genome low-coverage next generation sequencing has undertaken here for detecting small CNV and small balanced translocation.

## RESULTS

### Clinical report

**Figure 1:**
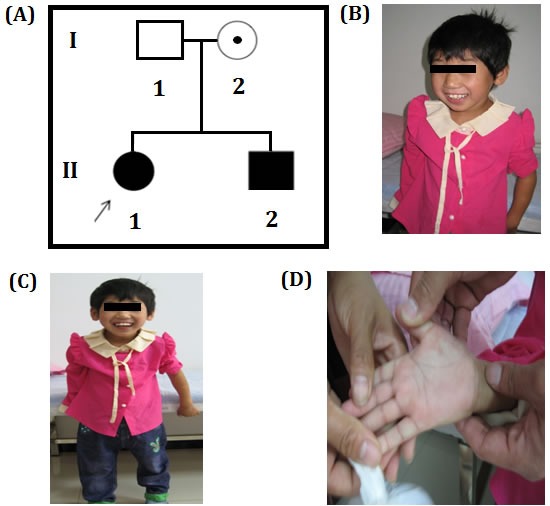
**A.** Pedigree of Chinese Family showing the proband and her little brother with intellectual disability. The filled symbol indicates the affected individuals, open symbol belongs to unaffected individuals, circle with a dot represents a asymptomatic carrier, square represents male and circles represents female. Arrow indicates the proband. **B.**, **C.** Chinese proband associated with intellectual disability and absence of speech, low level of understanding, wryneck, strabismus, and duck walking. **D.** Chinese proband's large and fleshy palms print pass through hand.

In this study, a Chinese family with Phelan–McDermid syndrome has recruited from Chenzhou, China. The proband was a 6 year girl with intellectual disability, absence of speech, unable to understand a few words, wryneck, strabismus, wadding gait, single transverse palmar crease. Proband's 3 year old younger brother also showed the similar clinical symptom. Proband is from a non-consanguineous family and her parents are phenotypically normal (Figure 1A-1D).

**Figure 2:**
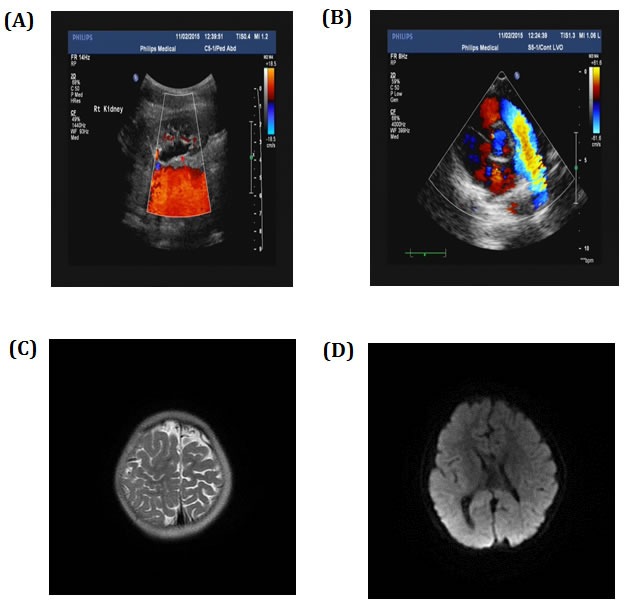
Ultrasonography showed **A.** double kidney effusion and **B.** normal heart. CT scan showed **C.** right side schizencephaly and **D.** callosal agenesis.

Considering the possibility of occurrence of brain and heart disease, we examined relevant organs. Ultrasonography test showed double kidney effusion (Figure 2A), heart was normal (Figure 2B). CT scan of brain showed right side schizencephaly (Figure 2C) and callosal agenesis (Figure 2D). The result of blood routine examination (the Complete Blood Count measures the number, variety, percentage, concentration, and quality of platelets, red blood cells, and white blood cells, and thus is useful in screening for infections, anemia, and other hematological abnormalities.), thyroid function test showed no abnormality. No biochemical and metabolic genetic diseases has been detected in proband and other family members in biochemical tests (include blood glucose, blood lipid, hepatic function, renal function tests) [Supplemental file S1].

Proband's 3 year old younger brother had the same clinical symptom like the proband, i.e. intellectual disability, absence of speech, unable to understand a few words, wryneck, strabismus, wadding gait, single transverse palmar crease. Proband and her younger brother had no history of perinatal asphyxia. Diagnosis of the patients has done by clinicians, clinical test reports and in order to understand the molecular basis of the disease in proband and her brother, chromosomes karyotype analysis and sequencing was performed.

**Figure 3:**
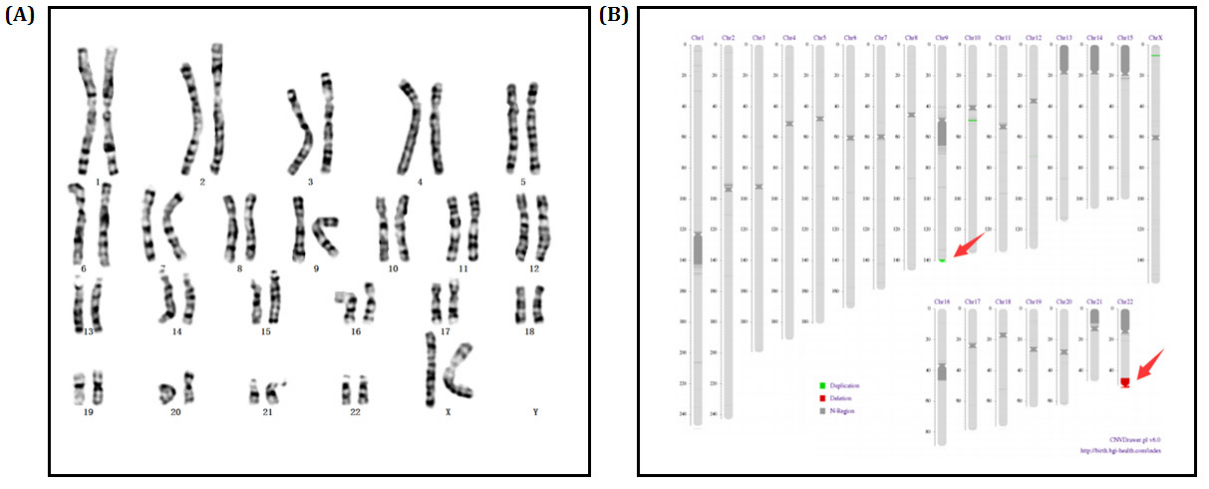
**A.** Chromosomes karyotype result, 46,XX of the proband (II-1). **B.** Electronic karyotype diagram of the Proband (II-1) Sequencing. “DUP” indicates “dupplication”; “DEL” indicates “deletion”; “BCA” represents balanced translocation; “INV” represents inversion; “INS” for insertion.

We screened the chromosome of proband (II-1) through karyotype and whole genome low-coverage sequencing. The karyotype result showed no abnormity (Figure 3A). However, sequencing detected an extra duplication variant on long arm of chromosome 9 besides the deletion variant on 22q13: 46, XX, dup(9q34.3). seq[GRCh37/hg19] (139,372,567-140,950,541)×3; 46, XX, del(22q13.31q13.33). seq[GRCh37/hg19] (44,882,702-51,146,914) ×1 (Figure 3B).

**Figure 4:**
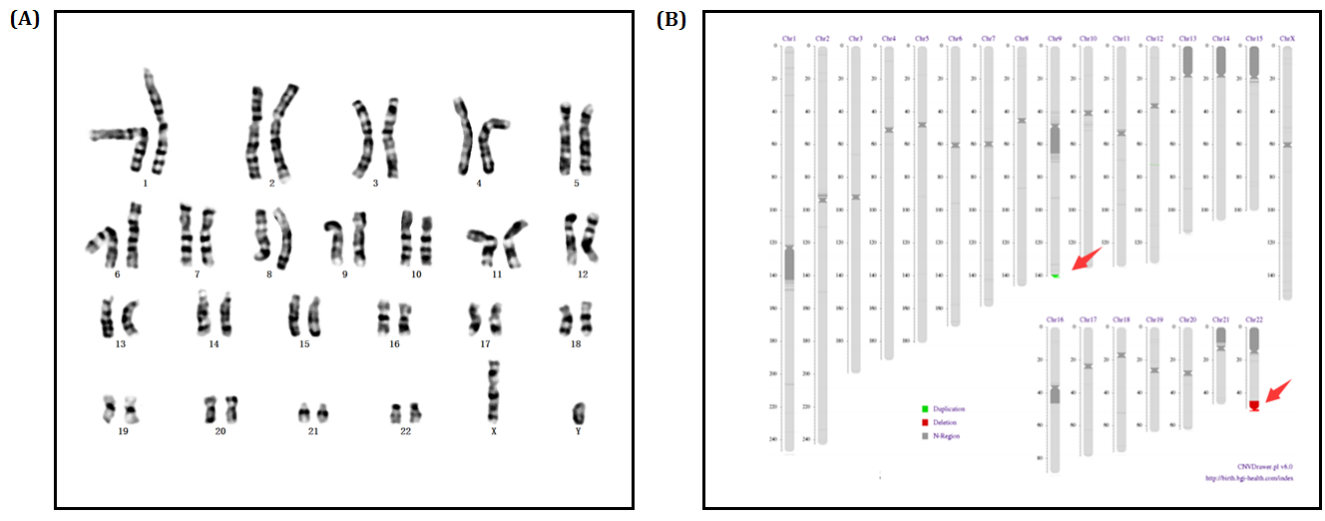
**A.** Chromosomes karyotype result, 46,XY of the Proband's little brother (II-2). **B.** Electronic karyotype diagram of the Proband's little brother (II-2) Sequencing. “DUP” indicates “duplication”; “DEL” indicates “deletion”; “BCA” represents balanced translocation; “INV” represents inversion; “INS” for insertion.

Since the clinical symptoms of proband's younger brother (II-2) was same as the proband, karyotype and sequencing was also performed to identify his genotype. Karyotype and sequencing result was almost the same to proband: 46, XY, dup(9q34.3). seq[GRCh37/hg19] (139,367,413-141,077,092)×3; 46, XY, del(22q13.31q13.33). seq[GRCh37/hg19] (44,882,702-51,146,914) ×1 (Figure 4A-4B).

**Figure 5:**
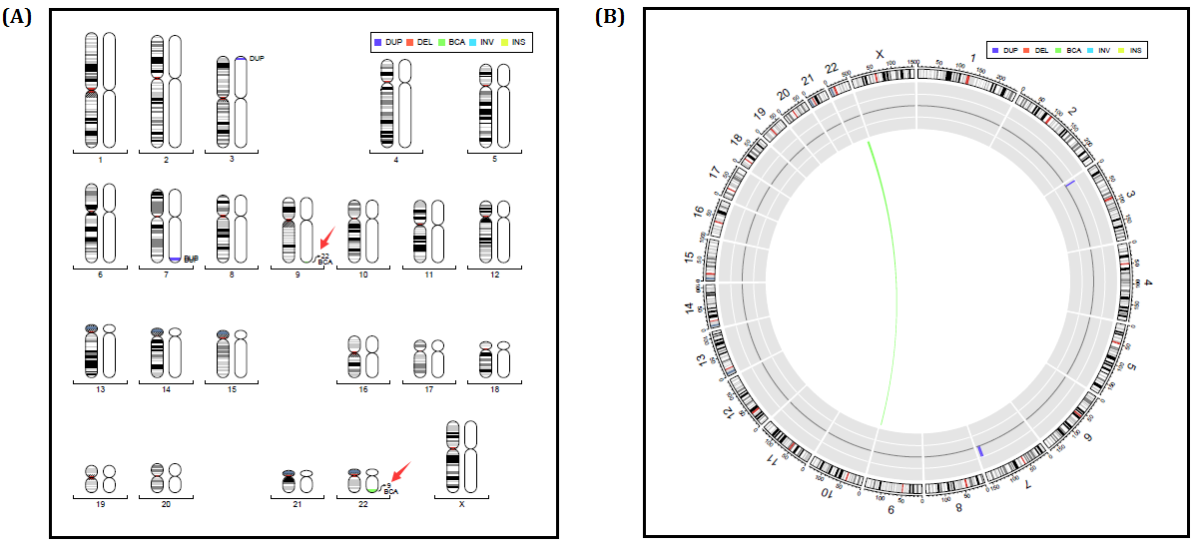
**A.-B.** Electronic karyotype diagram of the Proband's mother (I-2) Sequencing. “DUP” indicates “duplication”; “DEL” indicates “deletion”; “BCA” represents balanced translocation; “INV” represents inversion; “INS” for insertion.

In order to assess whether the parent is the carrier of translocation, whole genome low-coverage sequencing was performed for proband's parents. The karyotype of proband's father was normal and a translocation was detected on her mother: 46, XX, t(9;22)(q34.3;q13.31). seq[GRCh37/hg19] t(9;22) [(139,372,903-139,373,283); (44,909,509-44,909,776)] (Figure 5A-5B).

## DISCUSSION

In our study, we used karyotype and shallow whole genome sequencing to assess copy number variants and structural variants in this family. At first, karyotype failed to detect the small CNV on 9q34.3. In order to detect microdeletion or microduplication in the patients genome, low-coverage whole-genome sequencing was used in two children and identify 22q13 deletion syndrome patients with unbalanced translocation, which was due to a balanced translocation t(9;22)(q34.3;q13.31) from their mother. The deletion region of two children is partially overlap of 22q13 deletion syndrome (Phelan-Mcdermid syndrome), and it contained its major causative gene SHANK3 (Table 1).

**Table 1:** Genotype of Proband and all the family members

Affected Family Members	Sequencing result (GRch37/hg19)	Fragment size	Overlapping known syndrome	OMIM No.	Pathogenic genes included in OMIM
Proband (II-1)	46, XX, dup (9) (q 34.3), del (22) (q13.31 q13.33)	dup9:139372567-49358188, 1.58Mb; del 22.44882702-51146914, 6.26Mb	Partial overlap of 22q13 deletion syndrome	606232	SHANK3, NOTCH1, AGPAT2, MAN1B1, GRIN1, TPRN, SLC34A3, NELF, EHMT1
Proband's younger brother (II-2)	46, XY, del (22q13.31q13.33), dup(9q34.3)	dup9:139, 367, 413-141, 077, 092, 1.71Mb; del 22:44, 882, 702-51, 146, 914, 6.26Mb	Partial overlap of 22q13 deletion syndrome	606232; 251270; 250100	SHANK3; TUBGCP6; ARSA; ATXN10; ALG12; MLC1
Proband's mother (I-2)	46, XX, t(9;22) (q34.3; q13.31)	t(9;22) ((139, 372, 903-139, 373, 283); (44, 909, 509-44, 909, 776))	-	-	-
Proband's father (I-1)	46, XY	-	-	-	-

PMS is a common microdeletion syndromes (deletion 22q13.3) diagnosed over hundreds of individual worldwide (Phelan-McDermid Syndrome Foundation, Venice, FL). However, the diagnosis of PMS is challenging because the clinical symptoms are atypical, and overlapping with other chromosomal disorders. So, the accuracy of diagnosis requires the aid of the chromosomal testing.

It has been reported that 13 patients with varying sizes (95 kb to 8.5 Mb) of deletion in the 22q13.3 region also results in PMS [17]. In addition, a positive correlation between increased deletion size and varying clinical symptoms has been reported [3]. Larger deletions being associated with more severe phenotypes [16, 18] but some individuals with small deletions are also severely affected [4, 19, 20] Sarasua et al. identified clinical features associated with deletion size [21]. Previous reports suggest that there are clinically important genes located proximal to SHANK3 [16, 22].

However, according to the previous published reports, duplication of on long arm of chromosome 9 (9q34) leads to cause duplication 9q34 syndrome. Clinical symptoms associated with this syndrome are the severe dysmorphology of the hands and feet of children. In addition to arachnodactyly, the thumb and/or large toe may have abnormal implantation. Moreover, a *de novo* 9q34 gain in an infant with tetralogy of fallot with absence of pulmonary valve, with congenital heart disease in including the clinical symptoms of 9q duplication syndrome. But these clinical phenotypes are inconsistent with our proband. Additionally, the region of duplication of long arm of chromosome 9 for duplication 9q34 syndrome is larger than our patient's duplication region of long arm of chromosome 9.

The duplication region on the long arm of chromosome 9 in our present patient comprises of EHMT1, MAN1B1, NOTCH1, AGPAT2 and TPRN. None of these genes has reported with their duplications [23-26].

It is well known that conventional karyotyping (G-Band karyotyping) is the gold standard for the diagnosis of chromosomal disorders in clinical practice, .but karyotype analysis cannot always be characterised by scrutinising the G-banded pattern alone. Because its resolution is limited, microdeletions/microduplications (<5Mb) detection could not be applied. The recent reports implied that NGS technologies emerged, not only to achieve a quick scan of the whole genome, but also had prominent advantages of high accuracy, high-throughput, high sensitivity and low running costs, it has been widely used in medical diagnostics [27]. Next-generation sequencing has been reported to detect Balanced chromosomal rearrangement (BCA)-associated breakpoints with the aid of karyotyping. More recently, research reports demonstrated that low-coverage whole-genome sequencing has been applied to characterize chromosome structural variations with a high resolution, using different sequencing platform [28-31]. Our study results proved low-coverage whole-genome sequencing possesses significant advantages over conventional cytogenetic techniques once again.

It was obvious that low-coverage whole-genome sequencing and karyotyping techniques are very significant technology to diagnose PMS patients. The most important is low-coverage whole-genome sequencing providing a sensitive and accuracy detection platform for atypical chromosomal disorders in our study, it also provided optimal genetic diagnostic tool for clinicians.

In conclusion, we identified a novel deletion of 22q13.2q13.33 genomic regions associated with developmental delay, severely delayed speech, autistic like behaviors, and mild dysmorphic features. Our result expands the chromosomal deletion as well as the clinical phenotype associated with PMS syndrome.

## MATERIALS AND METHODS

### Patients and pedigree

In this study, a Chinese family with Phelan–McDermid syndrome has recruited from Chenzhou, China. The proband was a 6 year girl with intellectual disability and absence of speech. Proband's 3 year old younger brother also showed the same clinical symptom. Proband is from a non-consanguineous family and her parents are phenotypically normal. Proband and her younger brother had no history of perinatal asphyxia.

The Ethical Committee of the Chenzhou No.1 People's Hospital, China, reviewed and approved our study protocol in compliance with the Helsinki declaration. Family members of this five generation Chinese family have given written informed consent as they are participating in this study.

### Karyotyping

For 2 members, from peripheral blood lymphocyte cultures, chromosomes were prepared by following standard laboratory protocols. Cultured blood lymphocyte cell for 72 hours, transferred to centrifuge tube, discarded supernatant after centrifugation (8000 rpm, 10 min), conserved lymphocyte cell precipitation, hypotonic treatment for 30 min, immobilized chromosomes in acetic acid and formaldehyde mixed fixation fluid for 3 times, dropped 1-2 mL the chromosomes mixture to frozen slides, baked for 3-4hours in oven, pancreatin digestion for 30-60 seconds and Giemsa staining. By using standard protocols, analysis of G-banded chromosomes was carried out with a resolution of 550–850 bands per haploid genome.

### Whole genome low-coverage paired-end NGS

DNA samples from the proband, proband's younger brother and their parents were tested using a whole genome low-coverage paired-end NGS as described previously [32, 33].

Briefly, 3 μg of genomic DNA was sheared using HydroShear device (GeneMachine, San Carlos, CA) to construct a library with insert size of 3–8 kb. DNA fragments were end-repaired and 3’-end labeled with biotinylated nucleotides. After circularization via intramolecular ligation, they were then sheared again using the Covaris S2 sonicator to generate fragments of ~500 bp. These fragments were then purified using streptavidin-coated magnetic beads, end-repaired and A-tailed in preparation for ligation to paired-end oligo adapters. After adapter ligation, PCR was carried out using DNA fragments with adapter molecules at both ends. PCR products were size selected (~625 bp) by 2 % agarose gel electrophoresis. Libraries were subjected to 50-bp-end multiplex sequencing on the BGIseq-100 platform. After automatically removing adapter sequences and low-quality reads, high-quality pairedend reads were aligned to the NCBI human reference genome hg19 using the Short Oligonucleotide Analysis Package 2 alignment tool [34] (Table 2).

**Table 2:** Sequencing depth and the accuracy for calling a variant in low coverage shallow sequencing

Sample	Read_Length	Reads	Map_Rate	Sequencing Depth	Sequencing type
Proband (II-1)	49	24636014(24.64M)	96.18%	0.41	SE 50 Sequence
Proband's younger Brother (II-2)	49	27633974(27.63M)	96.01%	0.46	SE 50 Sequence
Proband's Mother (I-2)	50	60028505(60.03M)	78.84%	1	PE 50 Sequence
Proband's Father (I-1)	50	57645333(57.65M)	78.71%	0.96	PE 50 Sequence

### Detection of imbalanced chromosomal arrangements

DNA segments were generated from a sliding 50 kb window with 5 kb increments, and the mapped reads were classified into windows in terms of their mapped locations. The aligned location for each pair was set as the average value of the mapped locations of two ends. The coverage for each window was calculated as the mapped read pairs within its breakpoint locations. The coverage of each window was normalized to the mean value for all the windows in the experimental sample. The copy ratio of each window was calculated as the ratio of the corrected coverage to the mean coverage of that window across the control cohort. DNA segments identified by a circular binary segmentation algorithm [35] using corresponding copy ratios were defined as being deleted when the copy ratio was <0.75 or as being duplicated when the ratio was >1.25 [32]. Here, we only detected CNVs >100 kb because of low-coverage sequencing

### Detection of Translocations and Inversions

In principle, chimeric-read pairs should be observed in translocations and inversion events. These chimeric-read pairs suggested possible candidate BCA “clusters” throughout the genome. According to the insert size range setting for alignment, a small fraction of reads mapped with the mate-pairs at a distance or orientation that deviated from that expected based on the library insert size; this information was stored in a file named *.single. If viewed in the context of the previous studies [36], these read pairs would be flagged as being anomalous. For each sample, only this file was used as input for independent BCA discovery.

### Sequence analysis of junction fragments

The sequences of junction fragments were aligned to the human genome reference sequence (hg19) using Blast from NCBI. Analysis with the genomic context of the breakpoints was performed using the UCSC Genome Browser (http://genome.ucsc.edu/cgi-bin/hgGateway).

## SUPPLEMENTARY MATERIALS TABLE


