# Supplementary material for: Clinical and genomic evaluation of a Chinese patient with a novel deletion associated with Phelan–McDermid syndrome
Source: Oncotarget. 2016 Oct 10;7(49):80327–35. doi: 10.18632/oncotarget.12552 (PMC5348323; doi:10.18632/oncotarget.12552)
Supplement: Supplementary file 1 [file oncotarget-07-80327-s001.doc]

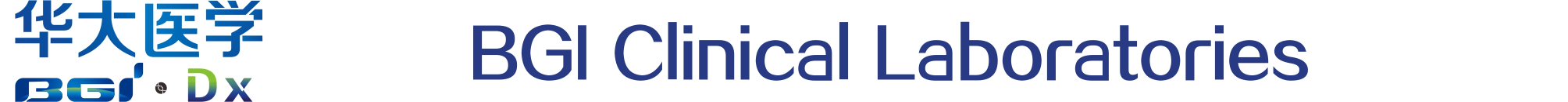


**Mass Spectrometry Testing Report for Neonatal Hereditary Metabolic Diseases**

**Sample Information**

| Father’s name:Proband’s father | Name of the subject: | Proband | Gender: | female |
| --- | --- | --- | --- | --- |
| Sample ID:15B6147095 | Tel: | Null |  |  |
| Date of Birth:Null | Date of blood collection: | 2015-11-02 | Test date: | 2015-11-10 |

Hospital/Institute:Chenzhou NO.1 People's Hospital

**Test Results**

| **Detection index** | **Detection index(abbr.)** | **Results** | **unit** | **lower limit** | **upper limit** |
| --- | --- | --- | --- | --- | --- |
| Alanine | Ala | 230.76 | µM | 50 | 400 |
| Arginine | Arg | 5.41 | µM | 1 | 40 |
| Aspartic Acid | Asp | 91.79 | µM | 10 | 150 |
| Citrulline | Cit | 18.87 | µM | 4 | 40 |
| Glutamine | Gln | 18.49 | µM | 2 | 60 |
| Glutamic acid | Glu | 320.53 | µM | 195 | 700 |
| Glycine | Gly | 310.26 | µM | 130 | 830 |
| Glutamic acid | His | 25.9 | µM | 4 | 135 |
| Leucine | Leu | 104.63 | µM | 50 | 250 |
| 2-amino-4-(methylthio)butanoic acid | Met | 25.08 | µM | 10 | 50 |
| L-Ornithine | Orn | 85.17 | µM | 15 | 235 |
| Phenylalanine | Phe | 61.58 | µM | 20 | 120 |
| Serine | Ser | 55.56 | µM | 20 | 210 |
| Threonine | Thr | 10.3 | µM | 8 | 50 |
| Tryptophan | Trp | 43.37 | µM | 20 | 80 |
| Tyrosine | Tyr | 53.39 | µM | 25 | 200 |
| Valine | Val | 106.7 | µM | 40 | 200 |
| Cit/Arg | Cit/Arg | 3.49 | ↑ | 0.25 | 10.00 |
| Gly/Ala | Gly/Ala | 1.34 |  | 0.90 | 6.00 |
| Gly/Phe | Gly/Phe | 5.04 |  | 3.00 | 15.00 |
| Leu/Phe | Leu/Phe | 1.70 |  | 1.10 | 5.00 |
| Met/Phe | Met/Phe | 0.41 |  | 0.25 | 1.00 |
| Orn/Cit | Orn/Cit | 4.51 |  | 1.50 | 18.00 |
| Phe/Tyr | Phe/Tyr | 1.15 |  | 0.20 | 2.00 |
| Free carnitine | C0 | 41.84 | µM | 11 | 65 |
| [Acetylcarnitine](http://www.baidu.com/link?url=dbAtQ-HVtgtL0zbU0ZV8lZER-mBldd3BoD18y5izXStsqGqRDXY26CXxVpER4YcjpvVVIZ53-4Webwm-ncI93z67cCBbKb0IfiKtWJhWvuwfywLQISXLfWLT7tSkOPMn) | C2 | 21.22 | µM | 8 | 50 |
| Propionyl-carnitine | C3 | 1.71 | µM | 0.4 | 4.5 |
| C3DC | C3DC | 0.05 | µM | 0.01 | 0.2 |
| C4 | C4 | 0.16 | µM | 0.05 | 0.6 |
| C4DC | C4DC | 0.57 | µM | 0.05 | 0.8 |

| **Detection index** | **Detection index(short)** | **Results** | **unit** | **lower limit** | **upper limit** |
| --- | --- | --- | --- | --- | --- |
| C4-OH | C4-OH | 0.1 | µM | 0.03 | 0.5 |
| C5 | C5 | 0.08 | µM | 0.04 | 0.4 |
| C5:1 | C5:1 | 0.01 | µM | 0 | 0.1 |
| C5DC | C5DC | 0.04 | µM | 0.01 | 0.2 |
| C5-OH | C5-OH | 0.1 | µM | 0.05 | 0.6 |
| C6 | C6 | 0.06 | µM | 0.01 | 0.2 |
| C6:1 | C6:1 | 0.02 | µM | 0 | 0.1 |
| C6DC | C6DC | 0.01 | µM | 0 | 0.1 |
| C8 | C8 | 0.07 | µM | 0.01 | 0.2 |
| C8:1 | C8:1 | 0.14 | µM | 0.02 | 0.45 |
| C8DC | C8DC | 0.02 | µM | 0 | 0.1 |
| C10 | C10 | 0.1 | µM | 0.01 | 0.25 |
| C10:1 | C10:1 | 0.14 | µM | 0.01 | 0.25 |
| C12 | C12 | 0.07 | µM | 0.02 | 0.5 |
| C12:1 | C12:1 | 0.02 | µM | 0 | 0.3 |
| C14 | C14 | 0.04 | µM | 0.05 | 0.5 |
| C14:1 | C14:1 | 0.07 | µM | 0.01 | 0.4 |
| C14-OH | C14-OH | 0.01 | µM | 0 | 0.1 |
| C16 | C16 | 0.68 | µM | 0.4 | 5 |
| C16:1 | C16:1 | 0.05 | µM | 0.02 | 0.45 |
| C16-OH | C16-OH | 0.01 | µM | 0 | 0.1 |
| C18 | C18 | 0.54 | µM | 0.18 | 2 |
| C18:1 | C18:1 | 0.92 | µM | 0.3 | 3 |
| C18-OH | C18-OH | 0.01 | µM | 0 | 0.05 |
| C10:2 | C10:2 | 0.01 | µM | 0 | 0.1 |
| C16:1-OH | C16:1-OH | 0.02 | µM | 0.01 | 0.15 |
| C18:1-OH | C18:1-OH | 0.01 | µM | 0 | 0.06 |
| C18:2 | C18:2 | 0.59 | µM | 0.06 | 0.8 |
| C18:2-OH | C18:2-OH | 0.01 | µM | 0 | 0.02 |
| C3/C0 | C3/C0 | 0.04 |  | 0.02 | 0.15 |
| C3/C2 | C3/C2 | 0.08 |  | 0.03 | 0.20 |
| C4/C2 | C4/C2 | 0.01 |  | 0.00 | 0.03 |
| C5/C2 | C5/C2 | 0.00 |  | 0.00 | 0.03 |
| C5DC/C8 | C5DC/C8 | 0.57 |  | 0.25 | 3.50 |
| C5-OH/C2 | C5OH/C2 | 0.00 |  | 0.00 | 0.02 |
| C5OH/C3 | C5OH/C3 | 0.06 |  | 0.01 | 0.35 |
| C5-OH/C8 | C5-OH/C8 | 1.43 |  | 0.70 | 12.00 |
| C8/C2 | C8/C2 | 0.00 |  | 0.00 | 0.01 |
| C14:1/C8:1 | C14:1/C8:1 | 0.50 |  | 0.10 | 4.00 |
| C0/(C16+C18) | C0/(C16+C18) | 34.30 |  | 3.00 | 50.00 |
| (C16+C18:1)/C2 | (C16+C18:1)/C2 | 0.08 |  | 0.05 | 0.40 |

**Result: No abnorm**[**it**](http://iask.sina.com.cn/c/1069.html)**y shown.**

**Analysis: No distinct hereditary metabolic diseases abnormality is found**

**Statement**

1、This report is specific to the tested sample, and cannot be used for other purposes.

2、Please consult the doctor of hospital to interpret the test results and diagnose diseases.

3、This test is used for 48 kinds of genetic metabolic diseases as follow, which can not be used for all metabolic diseases.

metabolic disorders of amino acid（Total:21）

1. maple syrup urine disease (2)Tyrosinemia type 1 (3)Tyrosinemia type 2 (4)Tyrosinemia type 3 (5)citrullinemia type 1(6)citrullinemia type 2(7)argininemia (8)hyperornithinemia (9)Nonketotic hyperglycinemia (10)ornithine transcarbamylase deficiency (11)Hypermethioninemia(12)N-acetylglutamatesynthetase (13)homocysteinemia (14)carbamoyl phosphate synthetase deficiency (15)argininemia(16)hyperammonemia hyperonithinemia homocitrullinuria (17)PKU(18)hyperhenylalaninemia(19)tetrahydrobioptein deficiency(20)histidinemia (21)hypervalinemia

[disorder of organic acid metabolism](http://www.baidu.com/link?url=r9SSbactVhIptCXuodJUgyxQEpLhJaGEg4cNWbcKvni9i_pkObIW5ygXJouEsTTuu8jOVsNxwjok9WBfX3uPJQblfF7LLI31qb_hMEzGDWk1rp732rqTTnAI5yalA0lW8VkLi13zqtRcNQAzZXpYi_) (Total:12)

(22)3-methylcrotonyl-CoA carboxylase deficiency (23)2- methyl coenzyme A dehydrogenase deficiency (24)propionic acidemia (25)isovaleric acldemla (26)3-methylglutaricacidemia (27)2-Methyl-3-hydoxybutryl CoA dehydrogenase deficiency (28)methylmalonicacidemia (29)Glutaric aciduria type 1 (30)Multiple carboxylase deficiency (31)Beta-Ketothiolase deficiency (32)Glutaric aciduria type 2 (33)3-hydroxy-3-methylglutaryl-CoA lyase deficiency

Fatty acid oxidation defect (Total:15)

(34)IBD deficiency (35)ethylmalonic encephalopathy (36)arnitine palmitoyltransferase I deficiency(37)arnitine palmitoyltransferase II deficiency(38)Short-chain acyl-coenzyme A dehydrogenase deficiency(39)Malonyl-CoA decarboxylase deficiency(40)medium chain acyl-CoA dehydrogenase deficiency(41)Long-chain acyl-coenzyme A dehydrogenase deficiency(42)carnitine deficiency(43)Functional protein deficiency(44)Carnitine-acylcarnitine translocase deficiency(45)medium-short chain acyl-CoA dehydrogenase deficiency(46)Very long-chain acyl-CoA dehydrogenase deficiency(47)medium chain 3-ketoacyl-CoA thiolase deficiency(48)2,4- coenzyme A reductase deficiency

Tested by: BGI Approved by: BGI Date: 2015-11-11
